# Voluntary Enhancement of Neural Signatures of Affiliative Emotion Using fMRI Neurofeedback

**DOI:** 10.1371/journal.pone.0097343

**Published:** 2014-05-21

**Authors:** Jorge Moll, Julie H. Weingartner, Patricia Bado, Rodrigo Basilio, João R. Sato, Bruno R. Melo, Ivanei E. Bramati, Ricardo de Oliveira-Souza, Roland Zahn

**Affiliations:** 1 Cognitive and Behavioral Neuroscience Unit and Neuroinformatics Workgroup, D’Or Institute for Research and Education (IDOR), Rio de Janeiro, Brazil; 2 Instituto de Ciências Biomédicas (ICB), Universidade Federal do Rio de Janeiro, Rio de Janeiro, Brazil; 3 Center for Mathematics, Computation, and Cognition, Universidade Federal do ABC, Santo André, Brazil; 4 Gaffrée e Guinle University Hospital, Federal University of the State of Rio de Janeiro, Rio de Janeiro, Brazil; 5 Centre for Affective Disorders, Institute of Psychiatry, King’s College, London, United Kingdom; Yale University, United States of America

## Abstract

In Ridley Scott’s film “Blade Runner”, empathy-detection devices are employed to measure affiliative emotions. Despite recent neurocomputational advances, it is unknown whether brain signatures of affiliative emotions, such as tenderness/affection, can be decoded and voluntarily modulated. Here, we employed multivariate voxel pattern analysis and real-time fMRI to address this question. We found that participants were able to use visual feedback based on decoded fMRI patterns as a neurofeedback signal to increase brain activation characteristic of tenderness/affection relative to pride, an equally complex control emotion. Such improvement was not observed in a control group performing the same fMRI task without neurofeedback. Furthermore, the neurofeedback-driven enhancement of tenderness/affection-related distributed patterns was associated with local fMRI responses in the septohypothalamic area and frontopolar cortex, regions previously implicated in affiliative emotion. This demonstrates that humans can voluntarily enhance brain signatures of tenderness/affection, unlocking new possibilities for promoting prosocial emotions and countering antisocial behavior.

## Introduction

In “Do Androids Dream of Electric Sheep?”, Philip Dick’s 1968 novel and later in Ridley Scott’s film “Blade Runner”, societal cohesion depended on the “empathy box” – an affiliative emotion-enhancing neurofeedback device. Although prospects of such a device remain in the realm of science fiction, recent advances in neuroscience and computer science have opened a window towards this possibility. Using neurofeedback approaches, individuals can gain access to their own brain activity and modulate it voluntarily. Affiliative emotion, including tenderness or affection, is underpinned by fronto-subcortical networks [1], [2]. Showing that these networks can be voluntarily modulated through neurofeedback would therefore provide a key step towards the realization of affiliative emotion enhancement. This would enable the development of novel interventions to enhance healthy psychological states and possibly to tackle antisocial and other maladaptive behaviors, which are often resistant to psychological, pharmacological and societal approaches [3]–[5].

Affiliative emotions (e.g., guilt, compassion, and tenderness/affection) depend on a frontopolar-septohypothalamic network [6]–[9] that is selectively engaged by affiliative compared with non-affiliative emotions such as anger/indignation, or disgust. Affiliative emotions are a key ingredient for moral behavior and empathy [10], [11], and were shown to activate the septohypothalamic area and the frontopolar cortex irrespective of positive or negative emotional valence [2]. Recent studies have demonstrated that functional MRI neurofeedback enables voluntary control of brain activation related to basic positive and negative emotions [12], [13]. Complex affiliative emotions, however, depend on more distributed activity in cortico-subcortical networks [11], [14]. It therefore remains unknown, whether individuals are able to use neurofeedback to modulate distributed neural networks underpinning such emotions.

**Figure 1:**
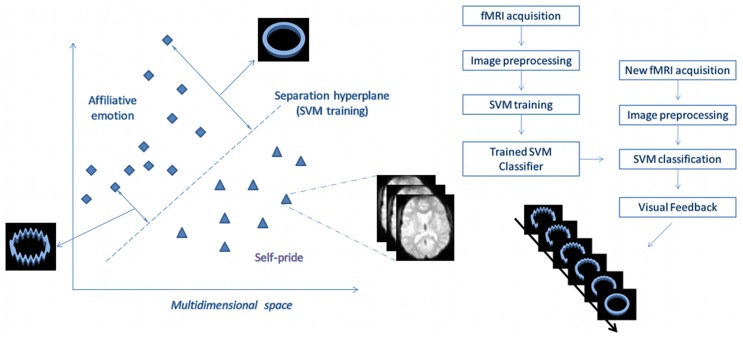
Graphic depiction of Support Vector Machine (SVM) algorithm used for classification and neurofeedback. The position of each diamond and triangle (representing brain volumes) relative to the decision boundary (multivariate separation hyperplane, dashed line) reflects the SVM classification of single brain volumes as a distributed activation pattern associated either with tenderness/affection or pride. The feedback stimuli comprised rings with different degrees of distortion (20 levels). The most distorted shape was associated with incorrect classification and the progressively smoother rings were associated with increasing distance of the correctly classified example from the SVM decision boundary.

Here, we used support vector machine-based (SVM) [15] brain decoding to provide multivariate real-time neurofeedback, implemented in a machine learning functional MRI analysis tool specifically designed for this purpose (see Methods). Participants were instructed to elicit specific autobiographical episodes evocative of strong positive emotions whilst undergoing functional MRI. In the affiliative condition they recalled life episodes associated with warm, tender/affectionate feelings involving friends or family [16]. In the pride control condition they recalled memories associated with pride related to own achievements, a complex positive social emotion involving the enhancement of one’s social status [17]. Participants were randomized into a neurofeedback and a non-neurofeedback group to control for neural changes related to repeated recall of emotional memories. The neurofeedback group received feedback through visual displays of rings whose degree of distortion reflected how characteristic their brain activation patterns were of tenderness when compared with pride (Figure 1). The control group saw randomly distorted rings that did not reflect their brain activation patterns. We hypothesized that neurofeedback facilitates attaining brain activation patterns distinctive for tenderness/affection. In addition, we predicted that neurofeedback training increases frontopolar and septo-hypothalamic hemodynamic responses to tenderness/affection.

## Materials and Methods

### Participants and Task Stimuli

Twenty-five healthy volunteers (14 women; age 24.5±3.5 years; education 16.9±2.8) took part in the study. One subject was excluded due to technical problems with MRI scanning. All participants were native Brazilian-Portuguese speakers with normal or corrected-to-normal vision, no history of psychiatric or neurological disorders, and none of them were taking centrally active medications.

Participants were randomly assigned either to a neurofeedback (NFB) or to a control (CTR) group. The NFB group performed the main tasks while receiving real-time fMRI visual feedback, while participants in the CTR group saw random stimuli. NFB and CTR groups were balanced according to age (NFB 23.8±0.7, CTR 25.2±1.2, *p = *0.3), gender (7 females and 5 males per group) and years of education (NFB 17.4±0.7, CTR 16.5±0.9, *p = *0.4). Written informed consent was obtained from all participants. The study was approved by the Ethics and Scientific committees of the D’Or Institute for Research and Education (Study #137/09).

Participants were interviewed by phone two days before the fMRI experiment and received a written guideline by email, which instructed them to carefully identify three different autobiographical episodes pertaining to the following categories: tenderness/affection, pride, and emotionally neutral. Specifically, participants were informed that the ‘tenderness/affection’ autobiographical scenario should be associated with positive emotions such as care, love and warm feelings towards a loved one (e.g., visiting a dear old friend). Importantly, tender/affectionate scenarios should not include sexual or romantic components (to increase specificity for tenderness/affection). For the pride scenarios, participants were instructed to retrieve an autobiographical episode involving own achievements and personal fulfillment, associated with positive feelings of pride in the presence of others (e.g., receiving public recognition for an accomplishment). Finally, the ‘neutral’ scenario should involve a trivial or ordinary episode devoid of any positive or negative emotions, but containing social components (e.g., shopping at the market). More details on task instructions and scenario generation can be obtained from the authors.

### Behavioral Scales and Questionnaires

Before and after MRI scanning, participants completed the PANAS (Positive and Negative Affect Scale) questionnaire [18]. Between each run of fMRI acquisition participants were asked to rate levels of tenderness/affection (intensity of tenderness felt in the tenderness condition), fatigue and focus during the experiment on 10-point Likert scales with anchor points 0 = “absent” and 9 = “very intense”. After the MRI experiment, participants also filled in a questionnaire to rate levels of emotional valence (15-point rating scales with anchor points −7 = “very unpleasant” and 7 = “very pleasant”, 0 = “neutral”), arousal (10-point rating scales with anchor points 0 = “absent” and 9 = “very intense”) and the vividness of visual imagery (VVIQ questionnaire; 5-point rating scale ranging from 4 = “perfectly clear and as vivid as normal vision” and 0 = “no image at all, you only ‘know’ that you are thinking of an object”) for each scenario. In addition, participants wrote a brief description of their scenarios.

### Task Procedure

The experimental task comprised four runs of approximately 10 minutes each. A long block-design was employed, and each emotional condition (4 tenderness/affection and 4 pride-related blocks per run; 22 volumes/44s for each emotional block) was interleaved with the neutral condition (8 blocks per run, 15 volumes for each neutral block) for disengagement from the previous task and signal normalization/detrending purposes. There were a total of 296 volumes per run. Before entering the scanner, participants had a preview of the experimental design through a computer presentation. For both tenderness and pride conditions participants were instructed to re-experience the emotion associated with their autobiographical episodes as intensely as possible. Participants were cued with a visually presented keyword to engage in the tenderness/affection, pride or neutral memories (“Neutral”, “Tenderness” and “Pride” [in Portuguese: “Neutro”, “Ternura” and “Orgulho”]). A custom-made software package (FRIEND [Functional Real-time Interactive Endogenous Neuromodulation and Decoding]; see http://fsl.fmrib.ox.ac.uk/fsl/fslwiki/OtherSoftware and http://idor.org/neuroinformatics/friend) was used [19] for real time fMRI data preprocessing (motion correction, spatial smoothing and normalization), feature selection and training of a multivariate classifier algorithm (Support Vector Machine, SVM). In the first run, the participant only saw the cues for the above conditions, without feedback. A two-class SVM was trained to discriminate between tenderness/affection and pride memories based on the distributed voxel patterns. In the three subsequent classification runs, the same design was employed, but this time combined with neurofeedback, represented by distorted rings. The most distorted ring shape was associated with patterns close to the SVM decision boundary hyperplane (i.e. indistinctive of the target emotion). Progressively smoother rings were associated with increasing distance of the SVM decision boundary (i.e. higher degree of distinction between tenderness and pride) (Figure 1). Real-time feedback was thus contingent on how well the current pattern of distributed brain activity (i.e., incoming image volume) matched the target defined during the previous runs.

Before the fMRI experiment, participants of the NFB group were informed that the visual feedback (rings of variable degree of distortion) they would see inside the scanner would reflect their ongoing brain activity while performing the task. The control group received control stimuli consisting of randomly distorted rings. The control group was aware that these rings were random. They were told that the rings were displayed to help them focus, and that their shape would be randomly changing. These participants were not aware of the existence of a neurofeedback group. Full instructions are provided in the Supporting Information. We purposefully chose not to employ sham feedback stimuli because this could lead to increased levels of frustration, thereby discouraging participants to keep engaged in the emotional tasks and compromising their performance.

### Image Acquisition

Functional images were acquired with a 3T Achieva scanner (Philips Medical Systems) using a T2*-weighted echoplanar (BOLD contrast) sequence (TR = 2000 ms, TE = 30 ms, matrix = 64×64, FOV = 240 mm, flip angle = 90°, voxel size 3×3 mm; slice thickness = 5 mm, 22 slices). Total functional scanning time was ≈ 55 min. Before each run, five dummy volumes were collected for T1 equilibration purposes. A SENSE factor of 1.5 and dynamic stabilization were additionally used. These parameters were based on careful sequence optimization to maximize temporal signal-to-noise [20] in brain regions that normally suffer from magnetic susceptibility effects, including the basal forebrain areas and ventromedial regions of the prefrontal cortex. High-resolution anatomical images were acquired with a 3D turbo field echo T1-weighted sequence (TR 7.1 sec, TE 3.4 sec, matrix 240×240, FOV 240 mm, slice thickness 1 mm, 170 slices). Head motion was restricted by using foam padding and straps over the forehead and under the chin.

### Neurofeedback Procedure and Multivariate Analysis

#### SVM methods

**Figure 2:**
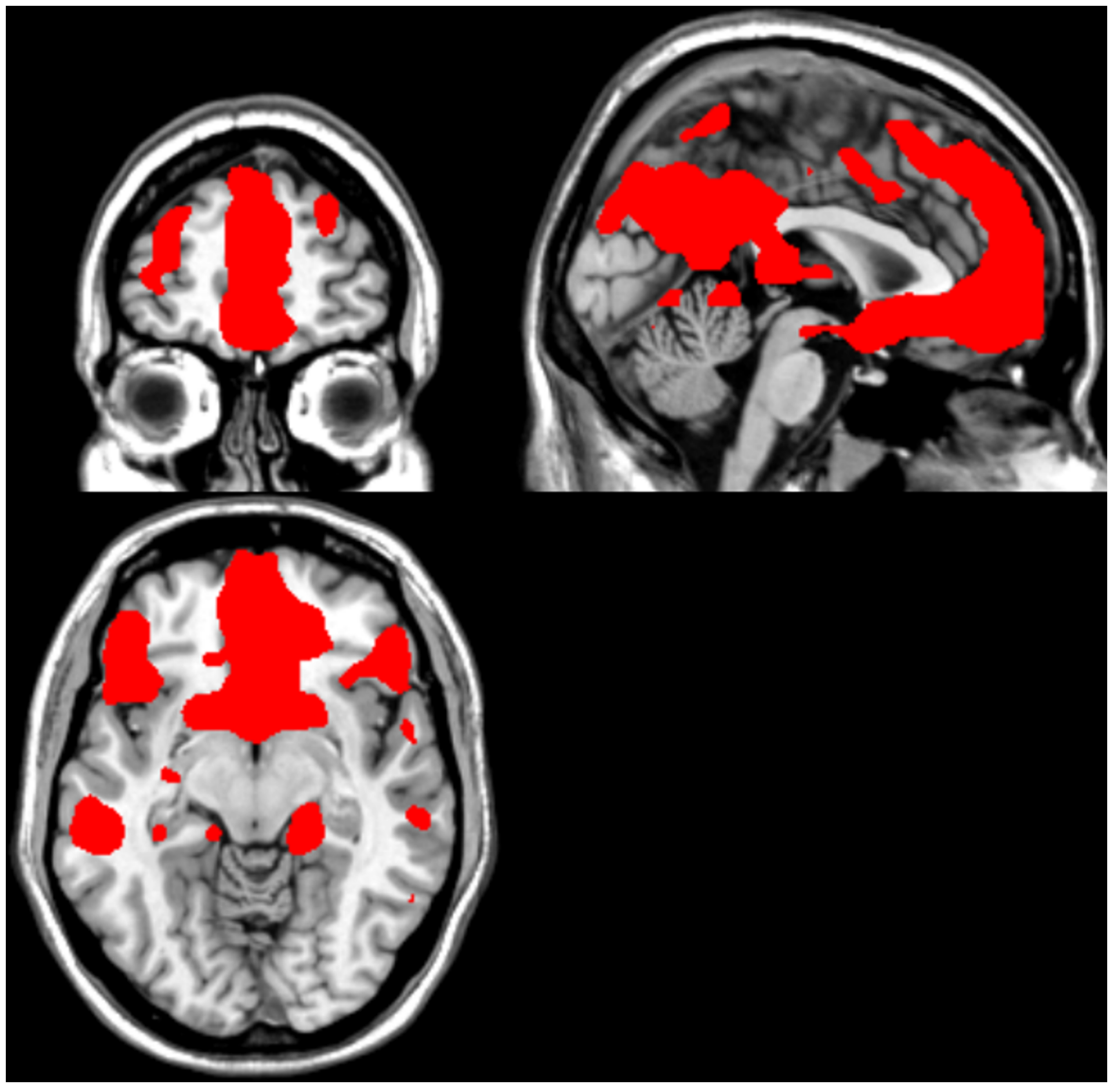
Feature mask employed for Support Vector Machine (SVM) input data. The mask includes sectors of the frontal, temporal, parietal and subcortical areas previously implicated in social emotions. The mask excludes brain regions involved in sensorimotor or visuo-spatial processing from SVM training and decoding.

A custom-made software [19] was used in order to process for motion correction (MCFLIRT algorithm), spatial smoothing (Gaussian Kernel, FWHM = 6 mm), GLM calculation, anatomically and functionally-defined ROI selection, automatic feature map generation and SVM training/classification. All steps described above employed native FSL codes [21], within a pipeline that enabled improved processing speed [19]. A two-class linear kernel SVM method was implemented in FRIEND and was used to encode patterns based on relative intensities of normalized signal of brain voxels across conditions and time. Signal-level normalization was performed by subtracting individual voxel signals averaged over the preceding neutral condition block from the current echoplanar images belonging to the tenderness/affection or pride condition block, which minimizes local signal trends. SVM training involved mapping input to output data for classification. Once this function is estimated (i.e., the classifier is trained), it can be used to obtain class prediction of new observations (see Figure 1) [22], [23]. In order to perform SVM training, the input data from selected brain voxels were rearranged into an input vector *x,* which was labeled according to the respective (ongoing) experimental condition. Subsequently, the projection of incoming fMRI image volumes onto a discriminating hyperplane was given by (*x^t^w+b*), where *w* is a vector containing the hyperplane coefficients and *b* is a constant. Once the signal was normalized, the rank of absolute coefficients values of vector *w* can be used to identify the most discriminant voxels [22]. A feature selection mask was employed to restrict SVM classification to sectors of the frontal, temporal and parietal cortices that are putatively important for social cognition and emotion, while excluding large sectors of the superior fronto-parietal cortex and posterior and ventral temporo-occipital cortices, in order to minimize contribution from brain regions involved in sensorimotor and visuo-spatial processing (details described below; see Figure 2).

#### Mask definition for feature selection

A feature selection mask was employed to restrict the input datasets for SVM training and decoding. This was done to exclude regions typically associated with visuo-spatial and sensorimotor processing. This mask derived from an independent study on brain responses to affiliative emotion [2], and was generated by the sum of three main contrasts (affiliative vs. non-affiliative, affiliative vs. neutral and non-affiliative vs. neutral, thresholded at *p* = .005, uncorrected). Adjacent striatal and basal forebrain regions putatively involved in tenderness/affection and/or pride were added to this mask (see Figure 2). The masks in MNI space were then warped to subject space by using the inverse transform of the FSL-FLIRT algorithm (affine, 12-parameters).

We chose not to use an SVM based feature selection approach to reduce dimensionality of the data in a whole brain analysis because the efficacy of feature selection in this context is still controversial [24]. Furthermore, we observed from pilot data using a similar design [19] that an automated feature selection algorithm did not provide improved results above and beyond a dimensionality reduction provided by a similar a priori mask that excluded visuo-motor regions and non-brain tissue.

#### SVM training pipeline

Data collection began with the acquisition of a high-resolution gradient-echo T1-weighted structural anatomical volume (reference anatomical image, RAI) and one high signal-to-noise echo-planar (EPI) volume (reference functional image, RFI), which were used as reference images. Functional images were then obtained using the real-time pipeline. The first two volumes of each block were excluded from SVM training and classification for BOLD signal stabilization purposes.

The training data (first run) was employed to train the classifier to discriminate between the experimental conditions of interest (tenderness/affection and pride). The trained SVM was then used in the subsequent brain decoding sessions, in which participants engaged in the same conditions of interest (but this time being presented either with contingent visual feedback or with random visual stimuli). At each new neurofeedback session, the previous run was used to re-train the SVM classifier. The order of condition blocks was balanced across the NFB and CTR groups (i.e., counterbalancing the experiment starting with either the tenderness or pride condition).

Because it is intrinsically difficult to estimate the amount of data necessary for a robust classification beforehand due to the complexity of the tasks, signal to noise ratios, implementation of the algorithms and other variables, we relied on extensive pre-studies using a similar SVM implementation and experimental designs of equivalent cognitive-emotional complexity (illustrated in [19]) in order to establish this parameter for the current investigation.

#### Real-time classification and neurofeedback

After training the SVM, predictions about the current type of activation pattern (tenderness/affection *vs.* pride) for the participant were made in real-time based on incoming fMRI image volumes. At this stage, neurofeedback-based modulation was accomplished through the presentation of visual feedback stimuli that were contingent on SVM classification.

Although SVM classification was based on categorical output data, linear SVM can provide the distance of a new observation to the separating hyperplane, the classification boundary between conditions [23]. This projection (“decision value”) was then used to determine the appropriate neurofeedback figure to be shown to the participant (i.e., a ring with a certain level of distortion; see Figure 1). Thus, these rings changed their shape every two seconds, from highly distorted to perfectly smooth according to the two-class SVM decision function values. The most distorted shape was associated with incorrect classification, and the progressively smoother rings were associated with increasing distance of the correctly classified example from the SVM decision boundary. Increasing distance from the SVM decision boundary indicated that the activation pattern was more distinctive of one condition as compared with the other.

### Off-line Analyses

#### Definition of ROIs for *a priori* hypothesis testing

**Figure 3:**
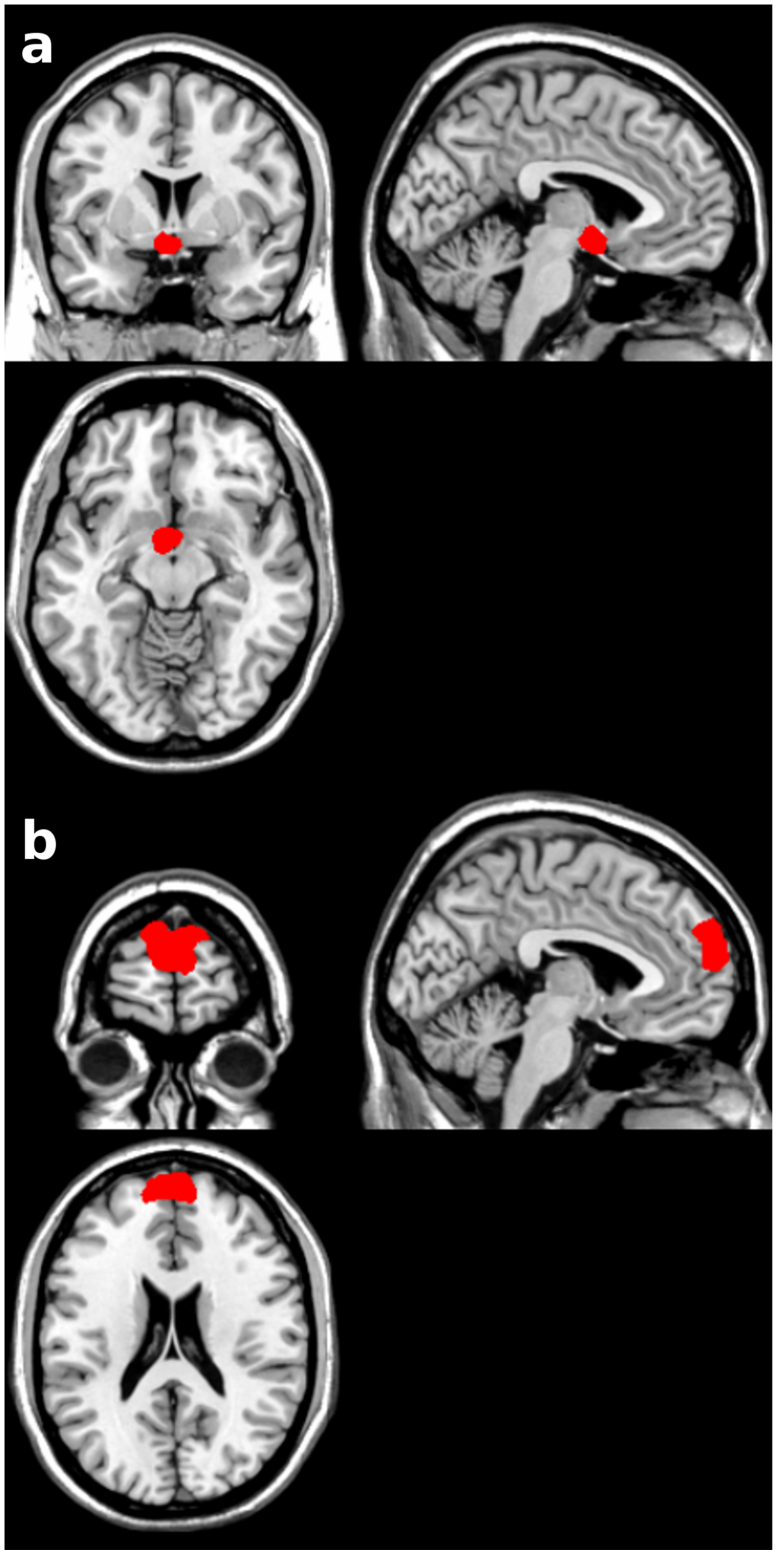
*A priori* anatomical masks. Septohypothalamic (a) and frontopolar cortex (FPC) anatomical masks (b) used for Small Volume Correction (SVC) in the univariate analyses of fMRI responses. These *a priori* masks contained 63 and 255 voxels (3×3×3 mm voxel size), respectively.

*A priori* ROIs of the septohypothalamic region and of the anterior prefrontal cortex were derived from an independent study [2] with further inclusion of the septohypothalamic region of the basal forebrain [25] and Brodmann’s area 10, encompassing the frontopolar cortex, FPC using MRIcron’s Brodmann’s area 10 ROI template (http://www.mccauslandcenter.sc.edu/mricro/index.html) (see Figure 3). These ROIs were employed for Small Volume Correction (SVC) for multiple comparisons (FWE) in Statistical Parametric Mapping (SPM8; http://www.fil.ion.ucl.ac.uk/spm/software/spm8/), for the purpose of univariate BOLD analyses described below.

#### Image preprocessing

SPM8 and the general linear model were used for offline BOLD image analysis [26], [27]. Pre-motion correction movement estimates per run (estimated root mean square [RMS] values for translation and rotation parameters) were restricted to 1.6. RMS mean value across runs. RMS values did not differ between groups (NFB = 0.52±0.06, CTR = 0.59±0.1, t [[22] = 0.5 p = 0.8, two-sample t-test). An additional control analysis showed that there were no significant correlations between movement estimates and BOLD effects or classification accuracy (see Supporting Information for details). Furthermore, echoplanar image (EPI) signal was sufficiently robust within the septohypothalamic mask in all participants, i.e., there were no significant EPI signal dropouts in this region (Figure S1). These analyses substantially reduced the possibility that motion or EPI dropouts could have substantially affected brain activation or classification accuracy estimates.

All functional datasets underwent registration and 12-parameter affine normalization to the standard MNI space (3×3×3 isotropic resolution). Functional data were smoothed using a 6 mm FWHM Gaussian spatial kernel. High-pass filtering (448 s) was used to remove unwanted low frequencies from the fMRI time series.

### Additional Statistical Analyses

Student t tests were applied for data showing a normal distribution (Shapiro-Wilk normality test). Non-normally distributed data were analyzed using non-parametric tests (Mann-Whitney and Kruskal-Wallis). Binomial tests were employed when appropriate. The alpha-level was set to p = .05, two-tailed.

### Univariate BOLD Analyses

For the within-subject, first-level analysis, the entire trial duration for each condition-specific block was modeled and convolved with a canonical hemodynamic response function [28]. Statistical effects were calculated on the second level by using a random effects model in SPM8. Analyses were performed by entering classification probability difference for tenderness (classification run 3– run 1) as a covariate in the design matrix. Significance was determined by using either a voxel-level FWE-corrected *p*<0.05 over *a priori* predicted regions of interest (Small Volume Correction; SVC) or at the whole brain level.

## Results

### Behavioral Results

Pre-scan levels of reported interest (*p* = .14, Mann-Whitney U = 48.5), excitement (*p* = .77, Mann-Whitney U = 67.0) or enthusiasm (*p = *.85, Mann-Whitney U = 68.5) did not differ between neurofeedback (NFB) and control (CTR) groups. There were also no differences in post-scan levels of interest (*p* = .44, Mann-Whitney U = 58.5), excitement (*p* = .98, Mann-Whitney U = 72.0) or enthusiasm (*p = *.57, Mann-Whitney U = 62.0) between NFB and CTR groups. Importantly, there were no overall differences on the reported levels of pleasantness (*p* = .76, Mann-Whitney U = 273.5) and arousal (*p* = .68, Mann-Whitney U = 268.0) between the tenderness/affection and pride scenarios across participants. Similarly, no difference was found on Vividness of Visual imagery Questionnaire (VVIQ) scores between conditions (*p* = .32, Mann-Whitney = 242.0). There was a main effect of time on fatigue level (*p*<. 0001, F = 14.3, two-way ANOVA). Feedback group had no effect on fatigue level (*p = *.60, F = .28), and there was no interaction effect between feedback group and time on fatigue (*p* = .77, F = .38). Besides, there was no interaction effect (*p = *.53, F = .73; group x time), as well as no main effect of either time (*p = *.85, F = .27) or feedback (*p* = .14, F = 2.37) on focus level of participants during the course of the experiment. Finally, participants reported overall high levels of tenderness during the experiment (NFB = 6.4±1.5; CTR = 6.3±1.5, before the first classification run; NFB = 6.6±1.4; CTR = 6.5±1.2, after the last run; scale maximum = 9), and between-run ratings of tenderness/affection (comparing levels before the first classification run with levels after the last run; two-way ANOVA) revealed no main effect of time (*p = *.70, F = .15) or group (*p = *.83, F = .05), and no interaction effect (*p = *1.00, F = .00).

### Brain Decoding Results

**Figure 4:**
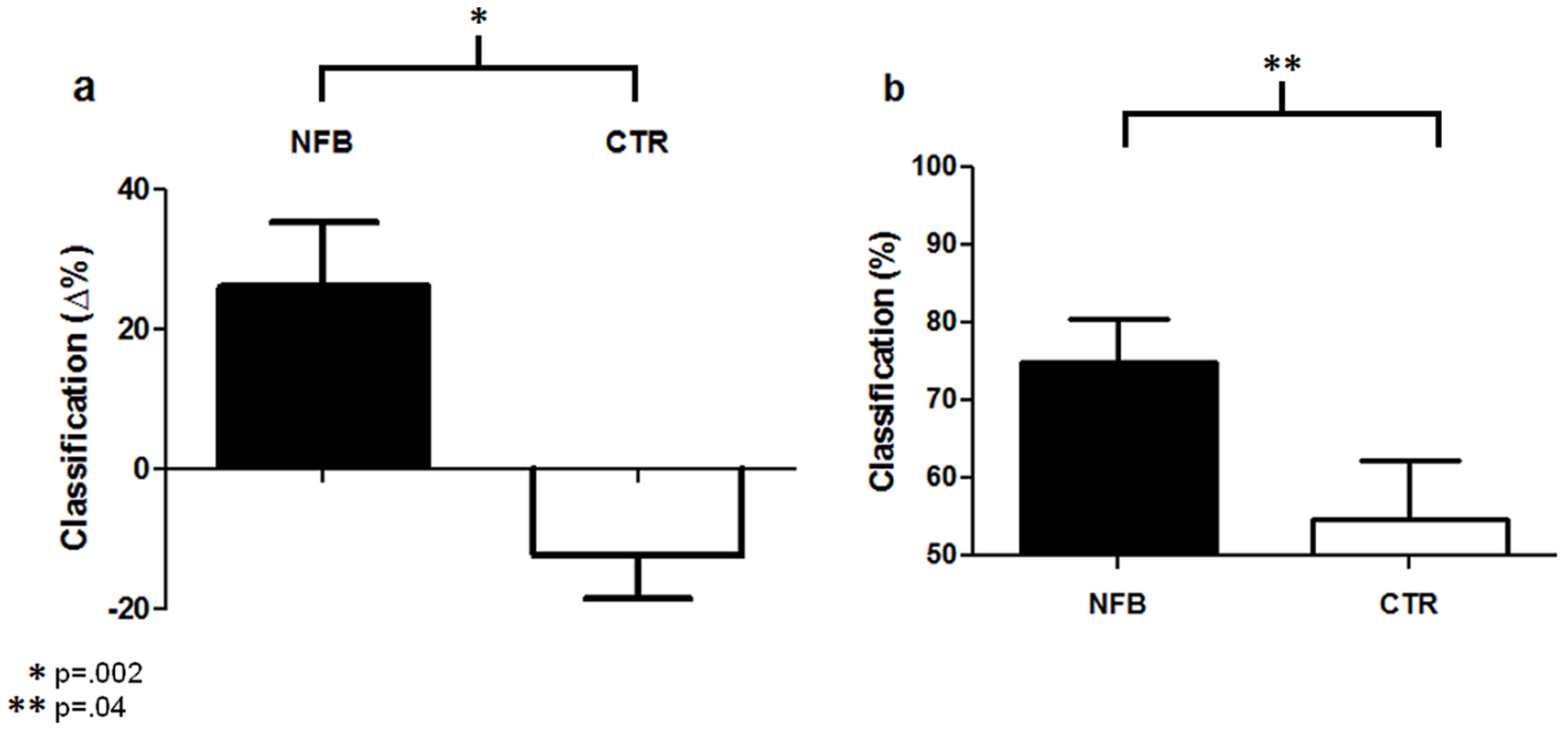
Support Vector Machine (SVM) classification results. (a) Difference in percentage of trials classified as characteristic of tenderness/affection between last and first fMRI sessions used for SVM classification. Results are shown for the neurofeedback (NFB) and the non-neurofeedback control group (CTR). (b) Percentage of trials classified as characteristic of tenderness at the last fMRI session (chance level = 50%).

**Figure 5:**
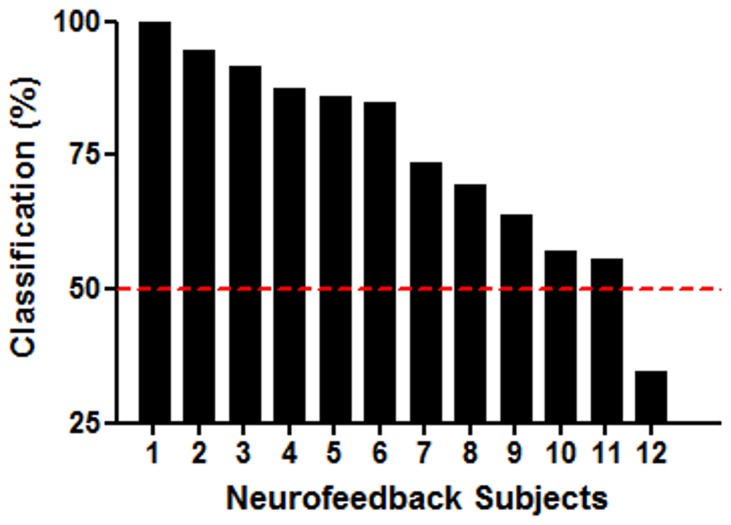
Percentage of trials correctly classified as characteristic of tenderness/affection for each participant of the neurofeedback group at the last classification run. Subject 12 spontaneously reported having changed the emotional elicitation strategy during the last run, possibly leading to the observed inverse classification pattern.

**Figure 6:**
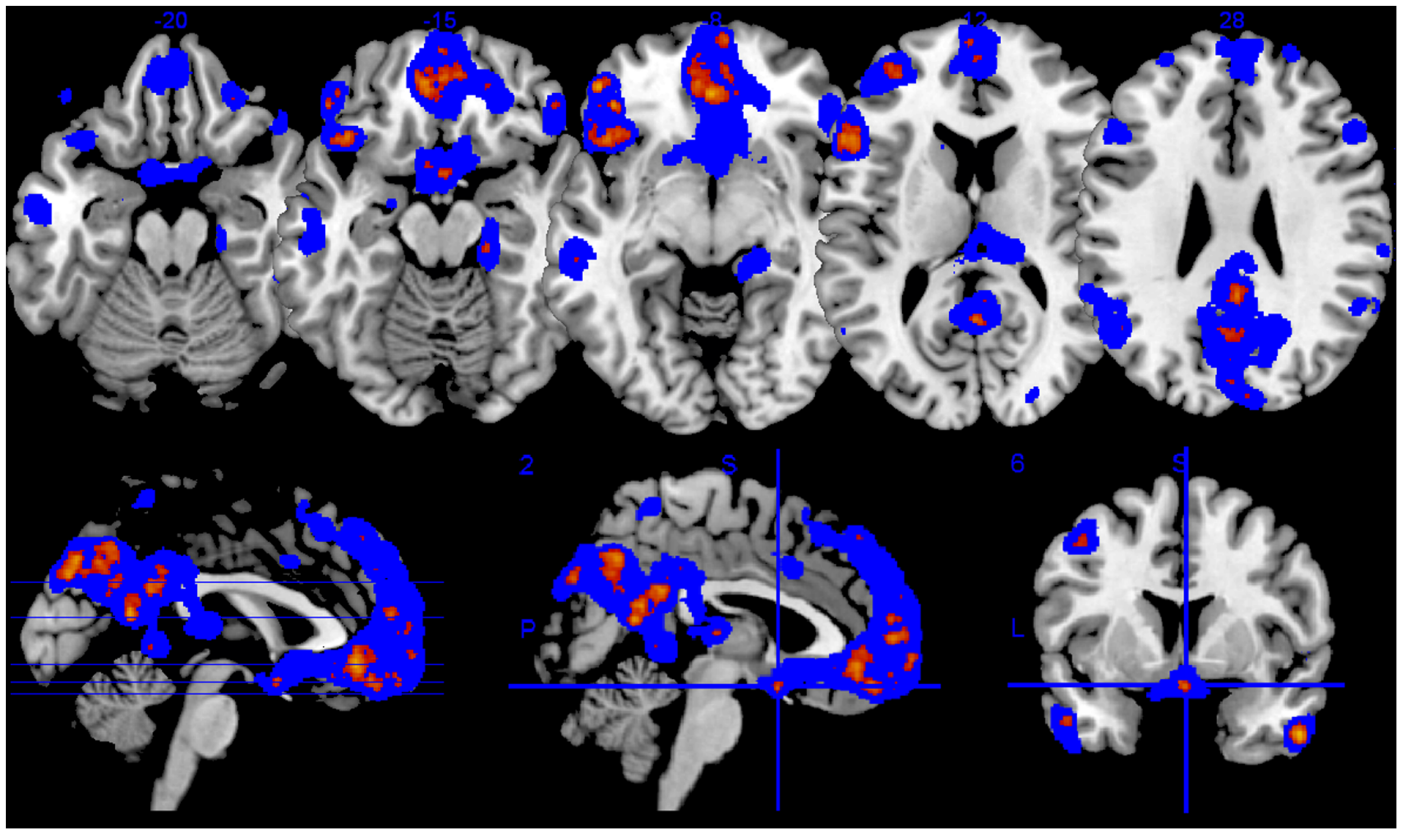
Discriminant voxels arising from multivariate pattern analysis. Distributed voxel patterns that best distinguished between tenderness/affection vs. pride within the neurofeedback group, measured across all fMRI sessions used for Support Vector Machine (SVM) classification. For display purposes, SVM weight maps were restricted to the 2% most discriminant voxels (highest absolute values of discriminant hyperplane coefficients). The color range shows voxels present in at least 40% (blue) or above 66% (red-yellow) of the subjects.

As predicted, neurofeedback significantly increased the percentage of trials of the affiliative condition classified as characteristic of tenderness/affection when comparing the last with the first classification session (mean difference = 26.0%, SD = 32.1%, t [11] = 2.81, p = .02). This effect was significantly different from the control group (t [22] = –3.4, p = .002) which did not show improvement, but rather a non-significant trend towards a decrease of the percentage of image volumes characteristic of tenderness/affection (mean difference = –12.3%, SD = 21.7%, t [11] = –1.96, p = .08, Figure 4a). In the neurofeedback group, the percentage of image volumes of the affiliative condition classified as characteristic of tenderness/affection in the last fMRI session was 75%±19.4% (t [11] = 4.44, p = .001; Figure 4b), and proved reliable in 9 of 12 participants (classifier performance exceeding 60% in these participants, p<.05, binomial test; Figure 5). In contrast, in the control group tenderness/affection-related brain activation patterns were classified only at chance level in the last fMRI session (54.6±26.1%; t [11] = .61, p = .50). This effect was significantly different between groups (t [22] = 2.15, p = .04). Figure 6 shows the distribution of brain voxels that best distinguished tenderness/affection from pride in the neurofeedback group. Additional analyses revealed that the spatial distribution of most discriminant voxels were similar across participants and runs, as well as between groups, suggesting that higher-order multivariate estimates – instead of more “simple” regional changes in brain activity – played a key role in the SVM classification results (see File S1 and Figure S2).

### Univariate Brain Activation Results

**Figure 7:**
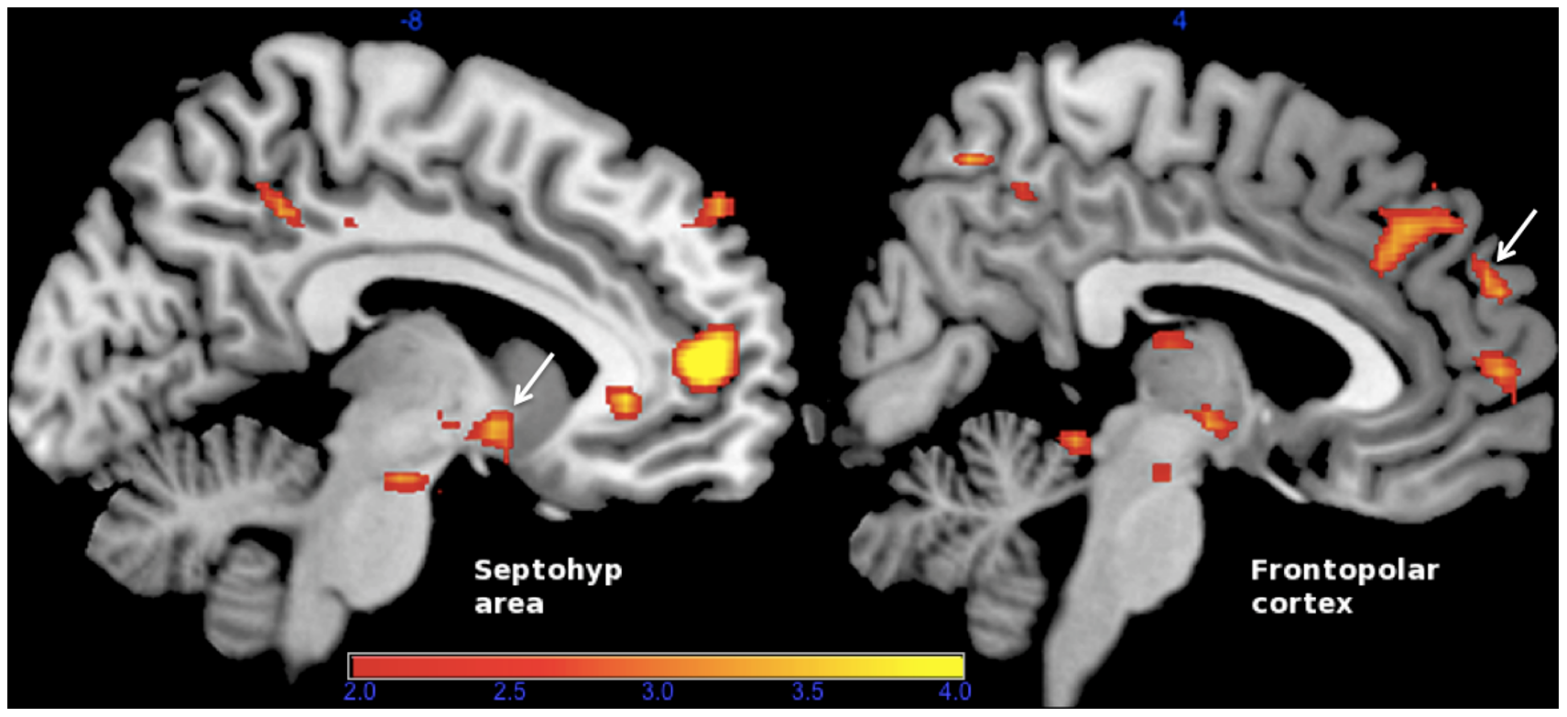
Regions of increased BOLD activity during neurofeedback training. Comparison of tenderness/affection *vs.* pride, using the change in frequency of attaining tenderness/affection-characteristic patterns observed in the last *vs.* first classification sessions as a covariate of interest. Significant effects (FWE-corrected) were observed in *a priori-*defined septohypothalamic (Septohyp) and frontopolar (FPC) regions of interest. Septohyp, MNI coordinates −6, 2, −11; cluster size (k) = 33 voxels; Z = 3.03, *p = *.046. FPC, MNI coordinates 3, 56, 22; k = 95; Z = 3.80; *p = *.016. All values are FWE-corrected using Small Volume Correction (SVC). There were no regions surviving FWE correction at the whole brain level.

We further investigated whether increased frequency in tenderness/affection-related brain activation patterns as determined by SVM in the neurofeedback group was associated with changes in regional Blood Oxygenation Level Dependent (BOLD) activity. For this purpose, we employed univariate analyses using the general linear model (GLM) approach [26]. The change in percentage of volumes belonging to the tenderness/affection condition that were classified as characteristic of this emotion, comparing the last to the first neurofeedback session, was computed for each participant and entered as a covariate of interest. As predicted, the probability of attaining brain activation patterns distinctive of tenderness/affection as compared to pride (i.e. the effect of neurofeedback training), was positively associated with BOLD responses in septohypothalamic and frontopolar regions (Figure 7). A supporting analysis ruled out that a simple scalar variable, namely mean image signal within the septohypothalamic ROI, could account for the SVM classification results (see File S1).

## Discussion

In this study we investigated whether participants can gain voluntary control over brain activation patterns related to affiliative emotion – more specifically, feelings of tenderness/affection, a cornerstone for prosocial moral behaviors, social attachment and emotional empathy [10], [29]. Our results demonstrate that multivariate brain decoding methods can be used to distinguish patterns of brain activation associated with tenderness/affection from another complex social emotion, achievement-related pride. We corroborated our first hypothesis that participants in the neurofeedback group were able to use brain decoding signals to enhance distributed brain activation patterns related to tenderness/affection. In contrast, a control group performing the same autobiographical emotional retrieval task but without the aid of neurofeedback failed to achieve brain patterns consistently classified as being distinctive of tenderness/affection. This was determined by comparing the number of trials classified as characteristic of tenderness/affection by the SVM algorithm at the beginning and the end of the experiment. Our second hypothesis was confirmed by showing that the training-related increase in probability of attaining a brain activation pattern characteristic of tenderness/affection observed in the neurofeedback group was associated with increases in septohypothalamic and frontopolar BOLD responses.

Our results provide the first demonstration that humans are able to voluntarily modulate the activity of a distributed ensemble of brain regions related to feelings of tenderness/affection. By using standard univariate analysis, we further demonstrated that neurofeedback training induced increased BOLD responses in key regions previously implicated in affiliative emotion. This result was obtained while using pride as an equivalently complex and socially relevant control emotion that had comparable levels of positive valence. Further, we were able to rule out that this result was due to repeated retrieval of emotional memories. This was ensured by comparing against a control group engaged in recall of emotional memories of equivalent content and intensity. Our results extend classical observations that humans are able to consciously modulate visceral activity and autonomic responses via biofeedback [30] insofar our participants achieved control over deep brain regions, including the hypothalamus. These findings also extend results from studies on brain decoding of basic emotional states such as disgust and happiness [12] by showing that more complex neural states associated with moral emotions can also be decoded and voluntarily controlled.

The offline processing of the fMRI data demonstrated that the networks engaged by tender/affectionate feelings are compatible with recent functional imaging and lesion studies addressing the neural bases of affiliative experiences, social cooperation, prosocial moral emotions and altruistic decisions [1], [6], [31], [32], [33]. Furthermore, we were able to distinguish between activated regions characteristic of tenderness/affection from those regions activated by pride, thus pointing to a selectivity of complex emotion representations. Whereas our SVM approach allowed us to test the contribution of largely distributed brain networks in guiding multivariate classification, the fact that brain regions implicated in affiliative emotion, such as the septohypothalamic area and the frontopolar cortex, were found to be part of the discriminant space provided further specificity to our findings.

Previous studies have shown a causal role of septal damage in predatory aggression and changes in sexual behavior in humans and animals [34]. In addition, stimulation of this region induces positive reinforcement and decreases intra-species aggression [35], [36]. Furthermore, the septohypothalamic region is a main binding site for oxytocin and vasopressin, which play key roles in social attachment [29], [37], [38], [39]. These subcortical structures are highly interconnected with cortical regions, including the frontopolar cortex [40], [41], which is known to enable sophisticated social cooperation [11], [29], [38], [42].

The experience of affiliative emotion is an important component of empathy [7], [10]. Empathic abilities include diverse mechanisms ranging from understanding another’s cognitive states (including intentions, emotions and beliefs) to responding to those with appropriate emotional responses such as compassion, guilt, shared joy and distress [43], [44], [45], [46]. Empathy is also involved in social decisions, emotional contagion, helping behaviors and group belongingness [47], [48], [49]. Our study investigated one specific affective facet of empathy, providing evidence that healthy participants may gain voluntary control over activity in brain regions that have been implicated in affiliative emotions in lesion and fMRI studies, in particular the septohypothalamic area and frontopolar cortex (Brodmann’s area 10) [6], [9], [32], [50], [51].

One central question for using fMRI neurofeedback is whether to employ feedback from single brain regions (using an ROI approach) or from multiple regions (e.g., using ROIs or SVM). Although we could have used single ROI neurofeedback from the septohypothalamic area, which has been implicated in affiliative emotion [2], we chose to use SVM because a number of additional regions, including the frontopolar cortex, were also part of this “affiliative network”. In addition, the comparison condition for our previous study (non-affiliative emotion), was not specifically associated with pride as in the present study, making direct comparisons across studies difficult. Furthermore, given our current understanding of the neural architecture of specific moral emotions, it is unlikely that activation in a single brain region would provide a specific real-time signature. Such complex emotions are associated with subtle differences in activity in several regions coding for action or event knowledge (prefrontal cortex), social conceptual knowledge (anterior superior temporal cortex), social sensory representations (posterior temporal cortex) and basic motivational and emotional states (subcortical structures) [11].

It should be emphasized that decoding of complex psychological experiences using multivoxel pattern analysis such as SVM relies on training and classification of distributed voxels. Thus, finding that a given experience, such as feeling tenderness/affection (as compared with pride), can be effectively decoded using SVM does not necessarily imply that the regions sheltering discriminant voxels are critical for the experience of these feelings. Thus, it was important to demonstrate that neurofeedback was associated with effects in regions previously shown to be critical for affiliative emotions, such as the septohypothalamic area. On a related vein, the possibility that non-specific brain responses in visuomotor regions might have contributed to the encoding and decoding of SVM patterns was minimized by (1) carefully instructing participants to select autobiographical scenarios endowed with similar features, emotional intensity and imageability (shown to be equivalent in the different conditions) and (2) by using an anatomical mask for classification that excluded visual and sensorimotor brain regions. One additional caveat is the possibility that neurofeedback led to increased attentional engagement and compliance with the task. Alternatively, control participants, who did not receive contingent feedback, might have found it difficult to keep engaged in the task or even be distracted by the random stimuli. This is a pervasive problem in neurofeedback studies, which by their very nature involve a degree of circularity. Nonetheless, reports of fatigue and focus across the fMRI experiment and pre vs. post scan levels of excitement, enthusiasm and interest did not differ between neurofeedback and control participants, making it unlikely that neurofeedback training effects were due to general attentional or motivational factors. Likewise, the finding that both NFB and CTR participants consistently reported elevated levels of tenderness/affection during the experiment confirms that they were suitably engaged in the emotional task. Thus, although we could not demonstrate that neurofeedback can increase the levels of tenderness/affection, our results do confirm a potential role of contingent neurofeedback in enhancing neural signatures of this emotional state. Further studies should address this point by employing more sensitive measures for affiliative emotions as well as appropriate measures for assessing transfer effects [12], [13]. Finding evidence of transfer effects of tenderness-related brain patterns longitudinally (e.g., see [52]) is a critical next step. Demonstrating transfer effects will also be crucial for the potential use of neurofeedback strategies in clinical settings (e.g., [53], [54]) – for example, promoting tenderness feelings in postpartum depression and antisocial personality disorder. The recent demonstration that an increase in whole-brain signal to noise can be attained by fMRI neurofeedback [55] supports the use of this approach to promote subtle and complex emotional states.

Future experiments may also explore the role of fMRI neurofeedback in enabling participants to control additional aspects of empathy and emotion, including empathic distress/contagion, inferring other’s psychological states and emotional learning [44], [56], [57]. The potential implications of being able to modulate neural patterns associated with diverse aspects of empathic responses are straightforward. For example, enhancing affection and compassion may counter dysfunctional social behaviors in specific contexts such as intergroup and couple relations [44] or disorders (e.g., psychopathy, conduct disorder).

Our findings build on a critical stream of advances in functional MRI instrumentation and data processing [58] that have enabled the development of real-time fMRI and neurofeedback [59], [60], [61], [62], [63], and multivoxel pattern analysis [64], [65], [66]. These developments enabled computer-intensive fMRI neurofeedback and classification of distributed patterns of brain activation to be simultaneously combined in real-time [12].

Taken together, our results demonstrate that neurofeedback can be used to enhance one’s ability to achieve distributed neural signatures of tenderness/affection. These findings represent an important step in the quest for the development of brain-machine interfaces allowing for voluntary modulation of the neural correlates of affiliative emotions. Allowing individuals conscious access to these neural processes could pave novel ways of fostering prosocial behaviors, by enhancing social attachment, trust, helping and care for others. This also opens up new possibilities, along with ethical issues, for the detection and treatment of disturbances of attachment across a range of neuropsychiatric conditions, including postnatal depression, psychopathy, and other personality disorders [4]. Future studies should address the next step towards this possibility by investigating whether repeated use of affiliative neurofeedback can enhance prosocial emotions and behavior in real-life settings.

## Supporting Information

Figure S1 **Anatomical coverage of the** ***a priori*** **region of interest (ROI) of the septohypothalamic region.** (a) The septohypothalamic ROI was used for small-volume correction for multiple comparisons, as well as for generating the mean echoplanar image (EPI) from all participants. The mean EPI image shows preserved signal at the basal forebrain (i.e., no signal dropouts at the individual and group level, except for a portion of the posterior orbitofrontal cortex). (b) Binarized EPI mask used by SPM8 at the second-level, overlaid on a T1 anatomical template. The septohypothalamic mask was also overlaid (in yellow), showing that it falls entirely within the areas of preserved EPI signal in all participants.
(TIF)

Figure S2 **Spatial distribution of the 2% most discriminative voxels in the brain across all runs of the experiment, for each experimental group (NFB and CTR).** Using the threshold above, the red-to-yellow color palette represents the percentage of participants contributing to this voxelwise effect (here thresholded at 66% of the subjects within each group). Thus, these maps reflect the inter-subject consistency of the most discriminative voxels contributing to the classifier. Visual inspection indicates stability of several voxels/regions across the experimental sessions (training run 1, classification runs 1–3). Direct comparisons across these multivariate-derived maps from different runs and groups using a voxelwise, univariate statistics is complex and would fall beyond the scope or goals of the present study. Figure S2 (a) NFB training run; (b) NFB classification run 1; (c) NFB classification run 2; (d) NFB classification run 3; (e) CTR training run; (f) CTR classification run 1; (g) CTR classification run 2; (h) CTR classification run 3. Note the similar spatial distribution of discriminant voxels across participants, runs and groups.
(TIF)

File S1 **Additional information concerning the methods section.**
(DOCX)
