# Supplementary figures and images for: Voluntary Enhancement of Neural Signatures of Affiliative Emotion Using fMRI Neurofeedback
Source: PLoS One. 2014 May 21;9(5):e97343. doi: 10.1371/journal.pone.0097343 (PMC4029815; doi:10.1371/journal.pone.0097343)

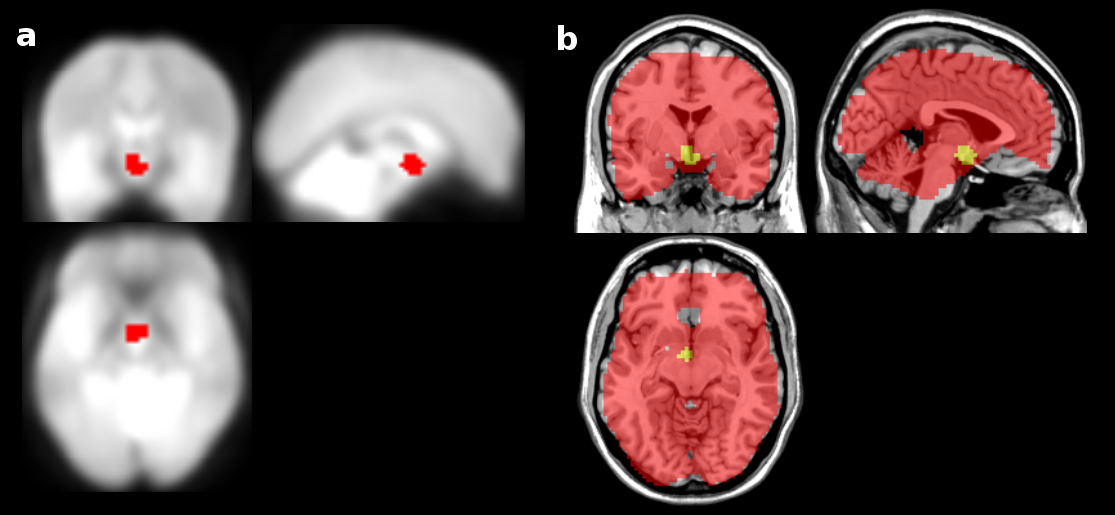

Supplement: Figure S1 — Anatomical coverage of the a priori region of interest (ROI) of the septohypothalamic region. (a) The septohypothalamic ROI was used for small-volume correction for multiple comparisons, as well as for generating the mean echoplanar image (EPI) from all participants. The mean EPI image shows preserved signal at the basal forebrain (i.e., no signal dropouts at the individual and group level, except for a portion of the posterior orbitofrontal cortex). (b) Binarized EPI mask used by SPM8 at the second-level, overlaid on a T1 anatomical template. The septohypothalamic mask was also overlaid (in yellow), showing that it falls entirely within the areas of preserved EPI signal in all participants. (TIF) [file pone.0097343.s001.tif]

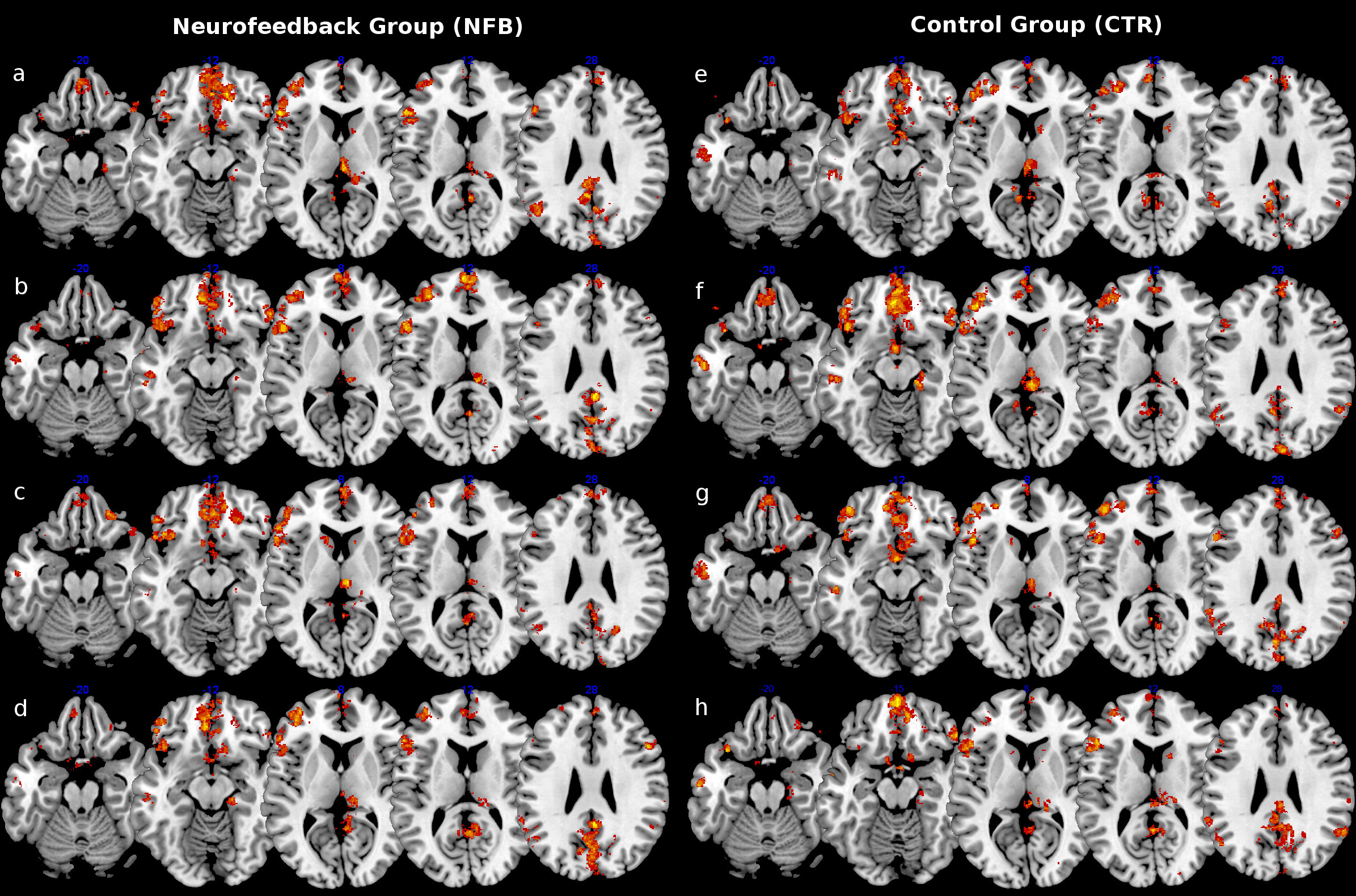

Supplement: Figure S2 — Spatial distribution of the 2% most discriminative voxels in the brain across all runs of the experiment, for each experimental group (NFB and CTR). Using the threshold above, the red-to-yellow color palette represents the percentage of participants contributing to this voxelwise effect (here thresholded at 66% of the subjects within each group). Thus, these maps reflect the inter-subject consistency of the most discriminative voxels contributing to the classifier. Visual inspection indicates stability of several voxels/regions across the experimental sessions (training run 1, classification runs 1–3). Direct comparisons across these multivariate-derived maps from different runs and groups using a voxelwise, univariate statistics is complex and would fall beyond the scope or goals of the present study. Figure S2 (a) NFB training run; (b) NFB classification run 1; (c) NFB classification run 2; (d) NFB classification run 3; (e) CTR training run; (f) CTR classification run 1; (g) CTR classification run 2; (h) CTR classification run 3. Note the similar spatial distribution of discriminant voxels across participants, runs and groups. (TIF) [file pone.0097343.s002.tif]
