# Supplementary material for: Voluntary Enhancement of Neural Signatures of Affiliative Emotion Using fMRI Neurofeedback
Source: PLoS One. 2014 May 21;9(5):e97343. doi: 10.1371/journal.pone.0097343 (PMC4029815; doi:10.1371/journal.pone.0097343)
Supplement: File S1 — Additional information concerning the methods section. (DOCX) [file pone.0097343.s003.docx]

**SUPPORTING INFORMATION**

**Voluntary enhancement of neural signatures of affiliative emotion using fMRI neurofeedback**

Jorge Moll^1*^, Julie H. Weingartner^1^, Patricia Bado^1,2^, Rodrigo Basilio^1^, João R. Sato^1,3^, Bruno R. Melo^1^, Ivanei E. Bramati^1^, Ricardo de Oliveira-Souza^1,4^, Roland Zahn^1,5^

*^1^ Cognitive and Behavioral Neuroscience Unit and Neuroinformatics Workgroup, D’Or Institute for Research and Education (IDOR) – Rio de Janeiro, Brazil*

*^2^ Instituto de Ciências Biomédicas (ICB), Universidade Federal do Rio de Janeiro – Rio de Janeiro, Brazil*

*^3^ Center for Mathematics, Computation, and Cognition, Universidade Federal do ABC – Santo André, Brazil*

*^4^ Gaffrée e Guinle University Hospital, Federal University of the State of Rio de Janeiro – Rio de Janeiro, Brazil*

*^5^ Centre for Affective Disorders, Institute of Psychiatry, King's College – London, United Kingdom*

*Corresponding author

Jorge Moll – *e*-mail: jorge.moll@idor.org

Cognitive and Behavioral Neuroscience Unit

D’Or Institute for Research and Education

Rua Diniz Cordeiro, 30/3° andar

Rio de Janeiro–22281-100, Brazil

Phone: [+55 21 3883-6000](tel:%2B55%2021%202538%203541)

*e*-mail: jorge.moll@idor.org

**Supporting methods**

*Task instructions to the neurofeedback group*

All the instructions were provided in Portuguese, the participants’ native language, and are here translated in English. “Once you enter the MRI scanner, it will be important that you remain completely still, because any minor movements can compromise the imaging data. You will be notified through the intercom when the task will begin. You must keep your eyes opened at all times. As the experiment begins, you will see the cue word for each emotional state displayed on the screen. As soon as this cue appears, you should start evoking the appropriate emotion (tenderness or pride, accordingly). There will be four sessions and between each session you will be asked some questions. In the first session you will see only the cue word followed by a fixation cross. Your task is to evoke the cued emotion as intensely as possible. In the three following sessions, you will see the cue word for each emotional state followed by rings that change in shape. The degree of distortion of the rings will reflect your ongoing brain activity. Between the emotional blocks (tenderness or pride) you will be cued to the emotionally neutral condition, during which a fixation cross will be displayed on the screen. Remember that your task is to experience the emotion as intensely as possible. The rings are present to help you in the task of eliciting the target emotions. A smoother ring indicates that you are on the 'right path’, so keep trying to feel the emotion intensely.”

*Task instructions to the control group*

Instructions to the control group were slightly different, as follows. “Once you enter the MRI scanner, it will be important that you remain completely still, because any minor movements can compromise the imaging data. You will be notified through the intercom when the task will begin. You must keep your eyes opened at all times. As the experiment begins, you will see the cue word for each emotional state displayed on the screen. As soon as this cue appears, you should start evoking the appropriate emotion (tenderness or pride, accordingly). There will be four sessions and between each session you will be asked some questions. In the first session you will see only the cue word followed by a fixation cross. Your task is to evoke the cued emotion as intensely as possible. In the three following sessions, you will see the cue word for each emotional state followed by rings that change in shape. The rings are displayed to help you focus and the degree of distortion of the rings will vary randomly. Between the emotional blocks you will be cued to the emotionally neutral condition, during which a fixation cross will be displayed on the screen. Remember that your task is to experience the emotions as intensely as possible.”

*Additional Control Analyses*

*Motion parameters:* We performed correlation analyses between motion estimates (root mean square of the 6 parameters; RMS) and the mean beta values of the main contrast (Tenderness vs. Pride) extracted from the septohypothalamic *a priori* mask. Correlations were not significant within either the NFB (r=.23, p=.18, R²=.05) or CTR group (r=.27, p=.11, R²=.07; all runs combined). Furthermore, no significant correlations were found between motion parameters and the percentage of trials correctly classified as characteristic of tenderness/affection (NFB group: r=-.11, p=.52, R²=.01; CTR group: r=.22, p=.21, R²=.05), when analyzing all runs combined. Similar results were obtained when analyzing each run separately (NFB group: [run1: r=-.13, p=.70, R²=.02; run2: r=-.32, p=.31, R²=.10; run3: r=.05, p=.90, R²=.002]; CTR group: [run1: r=.34, p=.27, R²=.12; run2: r=.30, p=.34, R²=.09; run3: r=.30, p=.38, R²=.08]).

*Echoplanar image (EPI) quality:* We have carefully assessed the possibility of T2* susceptibility artifacts in basal forebrain regions and took special care when checking individual data with respect to EPI signal and image distortion. Figure S1 (a) demonstrates the anatomical coverage of the *a priori* ROI of the septohypothalamic region, used for small-volume correction (SVC) for multiple comparisons, as well as the mean echoplanar image (EPI) from all participants. This mean EPI image shows preserved signal at the basal forebrain (i.e., no signal dropouts at the individual and group level, except for a portion of the posterior orbitofrontal cortex). Figure S1 (b) shows the binarized EPI mask used by SPM8 at the second-level, overlaid on the T1 anatomical template. The septohypothalamic mask was also overlaid (in yellow), showing that it falls entirely within the areas of preserved EPI signal in all participants.

*Exploring differences in signal magnitude across conditions:* We performed additional control analyses to determine whether the change in response/classification after neurofeedback could be more simply explained as function of differences in response magnitude. MRI signal magnitude within the septohypothalamic ROI belonging to the two emotional conditions, across the 3 classification runs, was compared within Neurofeedback (NFB) and Control (CTR) groups. There were no statistical differences in mean signal between the two conditions in the NFB (unpaired t-test, t[22]=.88, p=.39) or CTR (unpaired t-test, t[22]=.19, p=.85) groups, ruling out the possibility that a simple scalar value distinguished between these conditions.
